# Thymus Daenensis Extract and Essential Oils Effects on Morphine Withdrawal Signs in Mice

**DOI:** 10.17795/jjnpp-9959

**Published:** 2014-07-13

**Authors:** Mohammad Javad Khodayar, Esmaeil Taherzadeh, Amir Siahpoosh, Zahra Mansourzadeh, Seyed Amir Hossein Tabatabaei

**Affiliations:** 1 Toxicology Research Center, Department of Pharmacology and Toxicology, School of Pharmacy, Ahvaz Jundishapur University of Medical Sciences, Ahvaz, IR Iran; 2 Medicinal Plants and Natural Products Research Center, School of Pharmacy, Ahvaz Jundishapur University of Medical Sciences, Ahvaz, IR Iran; 3 Arvand International Division, Ahvaz Jundishapur University of Medical Sciences, Ahvaz, IR Iran

**Keywords:** Morphine, Substance Withdrawal Syndrome, Mice

## Abstract

**Background:**

*Thymus* species are well known medicinal plants which the previous studies suggested the involvement of the opioid system in them.

**Objectives:**

This study aimed to investigate the effects of methanolic extract and essential oil of aerial parts of *Thymus daenensis* (TD), an endemic aromatic medicinal plant of Iran, on morphine withdrawal syndrome in mice.

**Materials and Methods:**

Experiments were performed in two groups of five, each group treated with extracts or essential oils of TD. Dependency was induced by subcutaneous injection of morphine for three consecutive days. On the fourth day, the last dose of morphine was injected two hours prior to intraperitoneal injection of naloxone while the extract or essential oil of TD was administered 30 minutes before naloxone. A period of 20 minutes after naloxone injection was considered the critical period of the withdrawal syndrome. The number of jumps, standing, leaning, and the weight of stools were recorded as withdrawal signs.

**Results:**

The 200 mg/kg and 400 mg/kg doses of extract and all doses of essential oil decreased significantly the number of jumps, standing, leaning and the weight of stool. Administration of 100 mg/kg of extract only decreased the weight of stool and had no effect on the other factors.

**Conclusions:**

Extract and essential oil of TD attenuates morphine withdrawal behaviors in mice and may be useful in alleviating the signs and symptoms of opiate withdrawal syndrome in human.

## 1. Background

Addiction to drugs, including opioids, is a brain disease resulting in a loss of control over drug-taking or compulsive drug-seeking, despite its noxious consequences (1). Opioid dependence makes permanent physiological and psychological alterations leading to relapses of the disease. It is a serious social burden and has destructive influence on health and standard of living of an addicted person. Uncontrollable compulsion to take the drug and anxiety of craving and relapse are reasons of the opiates use. Efficient treatment protocols, and prevention of dependency development and addiction are not yet accessible (2).

Researchers identified the brain network which is responsible for mediating the reinforcing properties, rewarding pathways and craving phenomena of addictive drugs. The network involves such structures as the nucleus accumbens, ventral tegmental area, prefrontal cortex and limbic structures (3). Multiple targets exist for pharmacological intervention in opioid abusers. Opioid receptor antagonists are used for reversing overdose as well as antagonist-induced rapid detoxification during managed withdrawal (4).

 A separate strategy, usually following withdrawal and detoxification, is the treatment of addicts with pharmaceutical interventions primarily aimed at opioid maintenance therapy, usually with opioids such as methadone or buprenorphine. An important question is whether nonaddictive, non-opioid drugs, can satisfactorily substitute for maintenance therapy in treating opponent processes and craving. In this regard, treatment of addicts with non-opioid drugs, especially natural plant products represent another type of intervention (5).

*Thymus* species are well known medicinal plants. In traditional medicine, leaves and flowering parts of *Thymus* species are widely used as tonic and herbal tea, antiseptic, antitussive and carminative as well as treating cold (6-8). Thymus oil and extracts are widely used in pharmaceutical, cosmetic and perfume industry besides flavoring and preservation of several food products (9). It has been reported that naloxone reverses the antinociceptive and analgesic effect of *Zataria multiflora *and *Thymus vulgaris* (10, 11). This evidence indicated the role of opioid receptors in activity of *Thymus* species.

## 2. Objectives

Regarding the new therapies, the present study was designed to evaluate the methanolic extract and essential oil of aerial parts of TD on opioid withdrawal signs and symptoms.

## 3. Materials and Methods

### 3.1. Plant Material

The plant was collected during flowering stage from Yasouj, Iran, and identified in Medicinal Plant Research Center, Jundishapur School of Pharmacy, Ahvaz, IR Iran. The collected plant materials dried in shade under room temperature followed by grinding.

### 3.2. Preparation of Extract and Essential Oil

Powdered plants were extracted after 72 hours soaking in methanol. The extract was then concentrated under reduced pressure in a rotary evaporator to the desired volume. Solvent was removed using a freeze dryer (operon). The yield of the extract (dry powder) was calculated to be 5.7%. To prepare essential oil, dry aerial parts of TD were subjected to the water distillation. The content of essential oil yielded 2.2%.

### 3.3. Animals

Male N.MRI mice, weighted 25 ± 3 g, were obtained from the experimental animal house of Ahvaz University of Medical Sciences. All animals were maintained under controlled conditions of 25 ± 20˚C and 12 hours of light-dark cycles with free access to food and drinking water except during the experiments. All the ethical issues were considered based on the Ahvaz Medical University Ethical Protocols (AMUEP) on animal experiments.

### 3.4. Drugs

In this study, we used morphine sulfate ampoules (Darou Pakhsh pharmaceutical manufacturing company, Tehran, Iran), naloxone hydrochloride ampoules (Tolidaru pharmaceutical manufacturing company, Tehran, Iran) and diazepam hydrochloride ampoules (Chemidarou pharmaceutical manufacturing company, Tehran, Iran). The extract, essential oil and other drugs were diluted in normal saline and injected to the amount of 10 mL/kg. They were prepared immediately before use. The control groups received saline.

### 3.5. Induction of Dependency and Experimental Procedure

Dependency was induced based on the method of Zarrindast et al. (12). In brief, daily doses of morphine (50, 50 and 75 mg/kg) were injected subcutaneously (SC) for three consecutive days, and the tenth dose of morphine (50 mg/kg) was administered two hours before naloxone. All morphine-dependent mice were randomly divided into two groups of five mice as follows: group I, control mice; group II, diazepam (5 mg/kg) treated mice and groups III, IV and V, mice treated with different doses of extract (100, 200 and 400 mg/kg) or essential oil (0.5, 1, and 2.5 mL/kg). The route of administration of diazepam, essential oil and extract were intraperitoneally.

### 3.6. Naloxone-Induced Withdrawal Syndrome

Withdrawal syndromes were precipitated by an SC injection of naloxone (5 mg/kg). Then the animals were placed individually in an observation box. The number of jumps, the amount of wet and dry fecal material (weight of stool) and the number of standing and leaning were recorded immediately after naloxone injection over a 20-minute period. The diarrhea induced after naloxone administration was recorded as the weight in mg of fecal material/g body weight in 20 minutes.

### 3.7. Statistical Analysis

Analysis of variance (ANOVA) followed by Tukey test were used for comparisons of the data. Differences between means were considered statistically significant when P < 0.05. Each point is the mean ± SEM for eight mice.

## 4. Results

### 4.1. Effect of the Methanolic Extract and Essential Oil of TD on the Naloxone-Induced Jumping in Morphine-Dependent Mice

**Figure 1.**
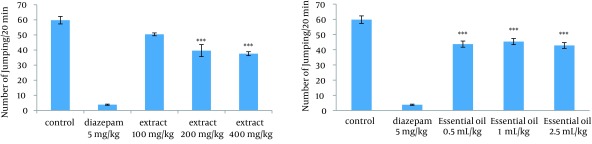
Effects of Different Doses of Extract (100, 200 and 400 mg/kg) and Essential Oil (0.5, 1, and 2.5 mL/kg) of TD on Jumping of Morphine-Dependent Mice Precipitated by Administration of Naloxone (5 mg/kg, IP) 2 Hours After the Last Dose of Morphine The jumping was used as an experimental index of withdrawal sign and counted during a 20- minute period. TD was administered IP 30 minutes prior to the naloxone. Values are expressed as Mean ± SEM. *** P < 0.0001 different from the respective saline control group.

Administration of 200 mg/kg and 400 mg/kg of extract and all doses of essential oil decreased significantly the number of jumps in morphine-dependent mice. The effect of extract was dose-dependent. As expected, diazepam also reduced the number of jumps in animals (Figure 1).

### 4.2. Effect of the Methanolic Extract and Essential Oil of TD on the Naloxone-Induced Diarrhea and Defecation in Morphine-Dependent Mice

**Figure 2.**
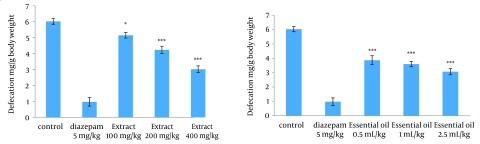
Effect of Different Doses of Extract (100, 200 and 400 mg/kg) and Essential Oil (0.5, 1, and 2.5 mL/kg) of TD on Defecation in Morphine-Dependent Mice Precipitated by Administration of Naloxone (5 mg/kg, IP) 2 Hours After the Last Dose of Morphine The defecation was used as an experimental index of withdrawal sign and counted during a 20-minute period. TD was administered IP 30 minutes prior to the naloxone. Values are expressed as Mean ± SEM. *** P < 0.0001 different from the respective saline control group.

Different doses of extract (100, 200 and 400 mg/kg) and essential oil (0.5, 1, and 2.5 mL/kg) of TD decrease the amount of defecation in morphine-dependent mice precipitated by administration of naloxone (5 mg/kg, IP) 2 hours after the last dose of morphine. The defecation was expressed as mg feces/gram body weight of the animal (Figure 2).

### 4.3. Effect of the Methanolic Extract and Essential Oil of TD on the Naloxone-Induced Standing and Leaning in Morphine-Dependent Mice

**Figure 3.**
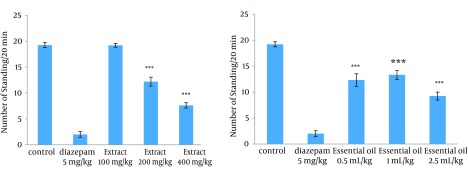
Effects of Different Doses of Extract (100, 200 and 400 mg/kg) and Essential Oil (0.5, 1, and 2.5 mL/kg) of TD on Standing in Morphine-Dependent Mice Precipitated by administration of Naloxone (5 mg/kg, IP) 2 Hours After the Last Dose of Morphine The standing was used as an experimental index of withdrawal sign and counted during a 20-minute period. TD was administered IP 30 minutes prior to the naloxone. Values are expressed as Mean ± SEM. *** P < 0.0001 different from the respective saline control group.

**Figure 4.**
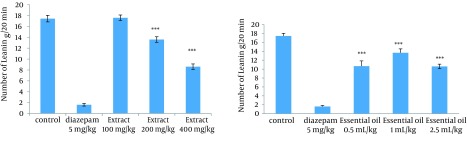
Effects of Different Doses of Extract (100, 200 and 400 mg/kg) and Essential Oil (0.5, 1, and 2.5 mL/kg) of TD on Leaning in Morphine-Dependent Mice Precipitated by Administration of Naloxone (5 mg/kg, IP) 2 Hours After the Last Dose of Morphine The leaning was used as an experimental index of withdrawal sign and counted during a 20-minute period. TD was administered IP 30 minutes prior to the naloxone. Values are expressed as Mean ± SEM. *** P < 0.0001 different from the respective saline control group.

The administration of extract in doses of 200 and 400 mg/kg, and all used doses of essential oil reduced significantly the number of leaning and standing (Figures 3 and 4).

## 5. Discussion

The present study examined the effect of TD on naloxone-induced behaviors such as jumping, defecation, standing and leaning (as withdrawal signs) immediately after injection over a 20-minute period. It seems that the potency of essential oil is more than the extract. However, the yield of essential oils was less than extract. All of used doses of essential oil alleviated the withdrawal signs in morphine-dependent mice. This suggests that lower doses of essential oils can also be helpful in reducing withdrawal signs in mice.

Nickavar et al. analyzed the essential oils obtained from the aerial parts of TD subsp. *daenensis* and reported twenty-six compounds, including thymol (74.7%), p-cymene (6.5%), β-caryophyllene (3.8%) and methyl carvacrol (3.6%) (13).

 Thymus essential oil and extract is a source of aromatic terpens, terpenoids, flavonoids and phenolic acids (14, 15). The flavonoids bind to GABAA receptors with different subtype specificity (16). The activities of flavonoids may be responsible for the alleviation of withdrawal signs in morphine-dependent mice. However, previous studies have reported that the mechanisms of some *Lamiaceae* plants such as *Rosmarinus officinalis* (17), *Nepeta glomerulosa* (18), *Salvia leriifolia* (19) and *Marrubium vulgare* (20) in reducing morphine withdrawal signs are mainly through the reduction of motor activity, as well as coordination and interaction with opioid receptors.

 Furthermore, reported spasmolytic activity of Thymus flavonoids may be evident in alleviation of defecation and diarrhea in this study (21, 22). In conclusion, our study showed that TD could alleviate withdrawal signs, and these activities can be related to the activity of extract and essential oils at central and peripheral sites. However, more studies recommended exploring the mechanisms of TD on alleviation of central and peripheral signs of the withdrawal signs and syndrome.
